# DCLK1 is a broadly dysregulated target against epithelial-mesenchymal transition, focal adhesion, and stemness in clear cell renal carcinoma

**DOI:** 10.18632/oncotarget.3059

**Published:** 2014-12-11

**Authors:** Nathaniel Weygant, Dongfeng Qu, Randal May, Ryan M. Tierney, William L. Berry, Lichao Zhao, Shweta Agarwal, Parthasarathy Chandrakesan, Harisha R. Chinthalapally, Nicholas T. Murphy, James D. Li, Sripathi M. Sureban, Michael J. Schlosser, James J. Tomasek, Courtney W. Houchen

**Affiliations:** ^1^ Department of Medicine, University of Oklahoma Health Sciences Center, Oklahoma, OK; ^2^ Department of Veterans Affairs Medical Center, Oklahoma, OK; ^3^ Peggy and Charles Stephenson Oklahoma Cancer Center, Oklahoma, OK; ^4^ Department of Cell Biology, University of Oklahoma Health Sciences Center, Oklahoma, OK; ^5^ Department of Pathology, University of Oklahoma Health Sciences Center, Oklahoma, OK; ^6^ COARE Biotechnology, Oklahoma, OK

**Keywords:** DCLK1, Renal Carcinoma, Tumor Stem Cell, Focal Adhesion, EMT

## Abstract

Renal clear cell carcinoma (RCC) is the most common type of kidney cancer and the 8^th^ most common cancer overall in the US. RCC survival rates drop precipitously with regional and distant spread and recent studies have demonstrated that RCC presents an epithelial-mesenchymal transition (EMT) phenotype linked to increased recurrence and decreased survival. EMT is a key characteristic of tumor stem cells (TSCs) along with chemo-resistance and radio-resistance, which are also phenotypic of RCC. Targeting these factors is key to increasing the survival of RCC patients. Doublecortin-like kinase 1 (DCLK1) marks TSCs in pancreatic and colorectal cancer and regulates EMT and stemness. Analysis of the Cancer Genome Atlas' RCC dataset revealed that DCLK1 is overexpressed and dysregulated on the mRNA and epigenetic level in more than 93% of RCC tumors relative to adjacent normal tissue. Immunohistochemistry using α-DCLK1 antibody confirmed overexpression and demonstrated a major increase in immunoreactivity in stage II-III tumors compared to normal kidney and stage I tumors. Small-interfering RNA (siRNA) mediated knockdown of DCLK1 resulted in decreased expression of EMT and pluripotency factors and significantly reduced invasion, migration, focal adhesion, drug-resistance, and clonogenic capacity. These findings suggest that DCLK1 is a novel, overexpressed factor in RCC progression that may be targeted to suppress EMT, metastasis, and stemness in early-stage and advanced RCC to increase patient survival. Moreover, the possibility that DCLK1 may mark a population of tumor stem-like cells in RCC should be further investigated in light of these findings.

## INTRODUCTION

Renal clear cell carcinoma (RCC) represents >90% of kidney and renal pelvis cancer, which is the 8^th^ most common cancer and represents approximately 4% of new cancer cases in the US each year [1, 2]. Each year 65,150 new cases are diagnosed and 13,680 people die from these diseases. Thirty-four percent of kidney and renal pelvis patients are diagnosed with regional (17%) or metastatic (17%) disease. Although the 5-year survival rate is 91.7% for patients with localized disease, patients with regional spread or metastatic disease have 64.2 and 12.3% 5-year survival rates respectively [1].

RCC is a highly radio- and chemo-resistant cancer characterized grossly by cell heterogeneity and hypervascularity [3, 4]. On the molecular level RCC is often associated with mutagenic or epigenetic inactivation of the Von-Hippel Lindau tumor suppressor gene (VHL) leading to altered expression of hypoxia-inducible and angiogenic factors [5, 6]. More recently, RCC has been shown to possess an epithelial to mesenchymal transition (EMT) phenotype and levels of EMT-related factors E-cadherin, Twist1, Vimentin, and β-catenin have been linked to adverse pathologies, increased recurrence, and reduced survival [2, 7].

Resistance to therapy, cell heterogeneity, and an EMT phenotype are key characteristics conferred by tumor stem cells (TSCs). Moreover, hypoxic conditions have been shown to support stemness in many cancers [8]. The stabilization of hypoxia-inducible factors as a result of VHL inactivation or direct somatic mutation in RCC may support an optimal environment for TSC-driven tumor progression. Doublecortin-like kinase 1 (DCLK1) is a tumor stem cell marker in colon cancer, pancreatic cancer, and likely other cancers [9-13] that is overexpressed in hypoxic conditions [13]. Lineage-tracing experiments using Dclk1-driven Cre recombinase mouse models demonstrate that the Dclk1+ tuft cell is a cell of origin for adenomas and colon cancer following loss of Apc in the intestine [9, 14]. Both the intestine and the proximal tubules of the kidney possess a prominent brush border, which aids in the process of nutrient reabsorption. Moreover, RCC arises from the proximal tubules in humans and can be initiated by loss of Apc in the renal epithelium in mice [15]. Despite these similarities and DCLK1′s emerging role as a TSC in gastrointestinal cancers, it has not been assessed in cancers of the excretory system. Here we demonstrate that DCLK1 is a potential therapeutic target against invasion, migration, focal adhesion, and stemness that is overexpressed in RCC tumors.

## RESULTS

### Patient Characteristics of the KIRC Dataset

The Cancer Genome Atlas' (TCGA) KIRC dataset was used to analyze patient mRNA, methylation, and protein levels. The average and median ages of patients upon diagnosis were slightly higher for females compared to males. There were almost twice as many males as females in the study, and the vast majority of the patients were white (>91%). Rates of distant metastasis for this dataset were similar to what has previously been reported [1, 16]. Approximately 1/3^rd^ of the patients had been reported deceased at the date linked to the dataset (Table SI).

### DCLK1 is overexpressed and has a unique methylation signature in RCC

**Figure 1:**
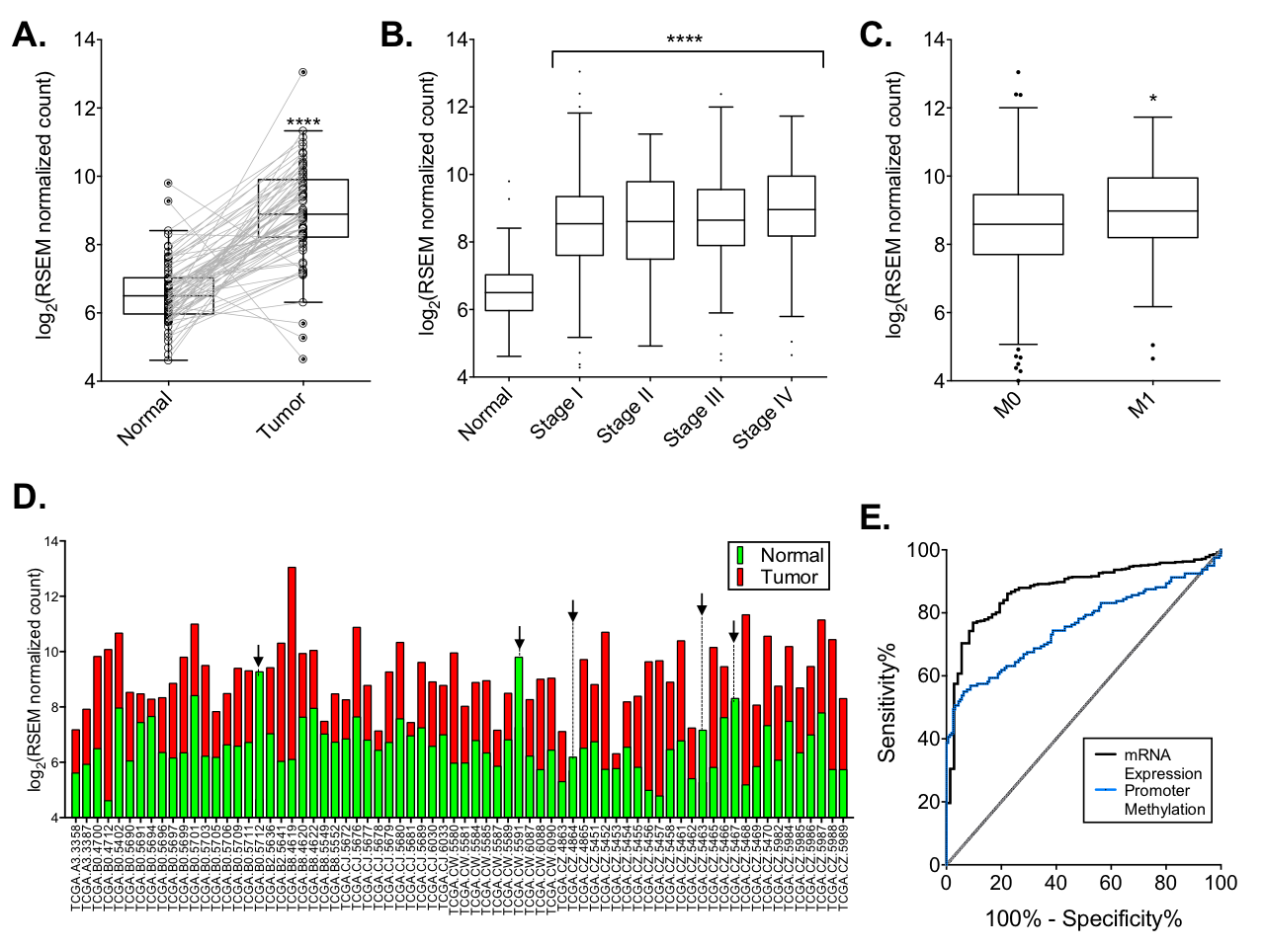
DCLK1 mRNA is overexpressed in the TCGA's clear cell RCC RNA-seq dataset A) DCLK1 mRNA is significantly overexpressed in patient tumors compared to matched adjacent tissue (p<0.0001). B) DCLK1 mRNA is consistently overexpressed in all stages compared to normal tissue (p<0.0001), but no stage-wise effects are present. C) DCLK1 mRNA expression is significantly increased in the primary tumors of patients with distant metastases (p=0.01). D) DCLK1 mRNA expression in tumor compared to matched adjacent tissue by individual patient. E) Receiver-operator characteristic curve for DCLK1 mRNA expression and promoter methylation in tumor tissue (p<0.0001).

Analysis of the TCGA KIRC RNA-Seq dataset [17] and multiple NCBI gene expression omnibus gene microarray datasets [16, 18] revealed that DCLK1 is massively overexpressed in RCC tumors compared to adjacent normal tissue (Fig 1A; Fig S1A-C). Additionally, the expression levels of DCLK1 were similar regardless of disease stage (Fig 1B). In patients with distant metastases DCLK1 was overexpressed approximately 1.3-fold (p=0.01, Fig 1C). Of the 72 patients in the KIRC RNA-Seq dataset with adjacent matched normal tissue only 5 patient tumors did not overexpress DCLK1 (Fig 1D). In order to assess whether DCLK1 mRNA expression may serve as a biomarker in RCC, we performed a receiver operating characteristic (ROC) curve analysis using Graphpad Prism 6.0. We found that DCLK1 mRNA expression is an excellent biomarker for RCC (p<0.0001, AUC = 0.876±0.019; Fig 1E).

**Figure 2:**
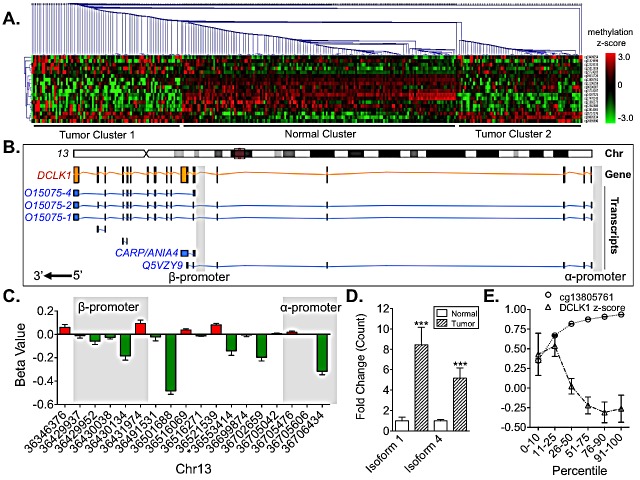
DCLK1 methylation is dysregulated and correlated to DCLK1 mRNA expression in the TCGA's RCC human methylation 450K dataset A) Single-linkage hierarchichal clustering demonstrating a unique DCLK1 methylation signature in tumor compared to matched adjacent tissue. B) Schematic of human DCLK1 chromosomal, gene, transcripts, and promoter locations based on the ENSEMBL database entry. C) Bar graph of individual DCLK1 methylation markers demonstrating a tendency towards hypomethylation in α- and β-promoter regions and overall. D) Isoform specific mRNA expression of DCLK1 isoforms 1 and 4 in normal compared to RCC tumor tissue. Isoform 1 is driven by the α-promoter and isoform 4 is driven by the β-promoter. E) Histone H3K27ac at Chr13:36553414 (HM450k probe cg13805761) is strongly associated with DCLK1 mRNA expression (p<0.0001).

Genes are often dysregulated in cancer as a result of epigenetic alterations, and global changes in tumor methylation are common [19]. Recently, DCLK1 methylation has been reported as a biomarker in cholangiocarcinoma and colon cancer [20, 21]. From 159 KIRC patients we compared tumor and matched normal samples that had been subjected to Illumina 450k Methylation Array analysis. We selected probes for analysis that demonstrated a p-value <0.001 between matched tissues by the Student's tTest. Using these probes we performed single-linkage hierarchical clustering and found that DCLK1 has a unique methylation signature in RCC tumor tissue (Fig 2A). DCLK1 has multiple promoters that produce multiple coding and non-coding transcripts (Fig 2B). A recent report focusing on a particular region of the DCLK1 α-promoter demonstrated hypermethylation in cholangiocarcinoma suggesting gene knockdown and a potential tumor suppressor function for DCLK1 in that cancer [20]. We found that although part of this region was hypermethylated in RCC, when the promoter as a whole is taken into consideration strong hypomethylation is present. Moreover, we found that the β-promoter was also hypomethylated (Fig 2C). ROC curve construction and analysis revealed that DCLK1 promoter methylation can serve as a biomarker in RCC (p<0.0001, AUC = 0.758±0.028; Fig 1E). To determine which promoter's methylation was a better biomarker we constructed separate ROC curves for each promoter and used the formula

$$z=\frac{AUC_{1}-AUC_{2}}{\sqrt{(SE_{1}{}^{2}}-SE_{2}{}^{2}-2rSE_{1}SE_{2}}$$

where *AUC* is the area under the curve, *SE* is the standard error, and *r* is the Hanley-McNeil Coefficient obtained by correlating the average Kendall Tau Correlation Coefficient between normal samples and tumor samples

$$\frac{\tau N+\tau T}{2}$$

and the average *AUC* [22]. Using this method we found that β-promoter methylation (AUC = 0.838±0.024) was a significantly better biomarker than α-promoter methylation (AUC = 0.629±0.032) with *z* = −5.345 where *z* above 2 or below −2 is considered to be statistically significant.

Next we assessed isoform specific RNA-Seq data from these same patients and found that isoform 1, which is produced from the α-promoter, and isoform 4, which is produced from the β-promoter, are both significantly overexpressed (Fig 2D). CAMK-related peptide (CARP/ANIA4), another product of the β-promoter also demonstrated increased expression, but was not statistically significant (data not shown). Isoform 3 was not expressed in either normal or tumor tissue in the kidney, save for a few samples at low levels (data not shown). Additionally, we assessed the correlation between methylation of probe cg13805761, which is associated with an intronic region with high Histone 3 Lysine 27 activity at Chr13:36553414 according to ENCODE [23], and DCLK1 mRNA expression as reported in the Broad Institute's standard data analyses [24]. Our analysis confirmed this relationship (Pearson R = −0.2498, p<0.0001; Fig 2E).

**Figure 3:**
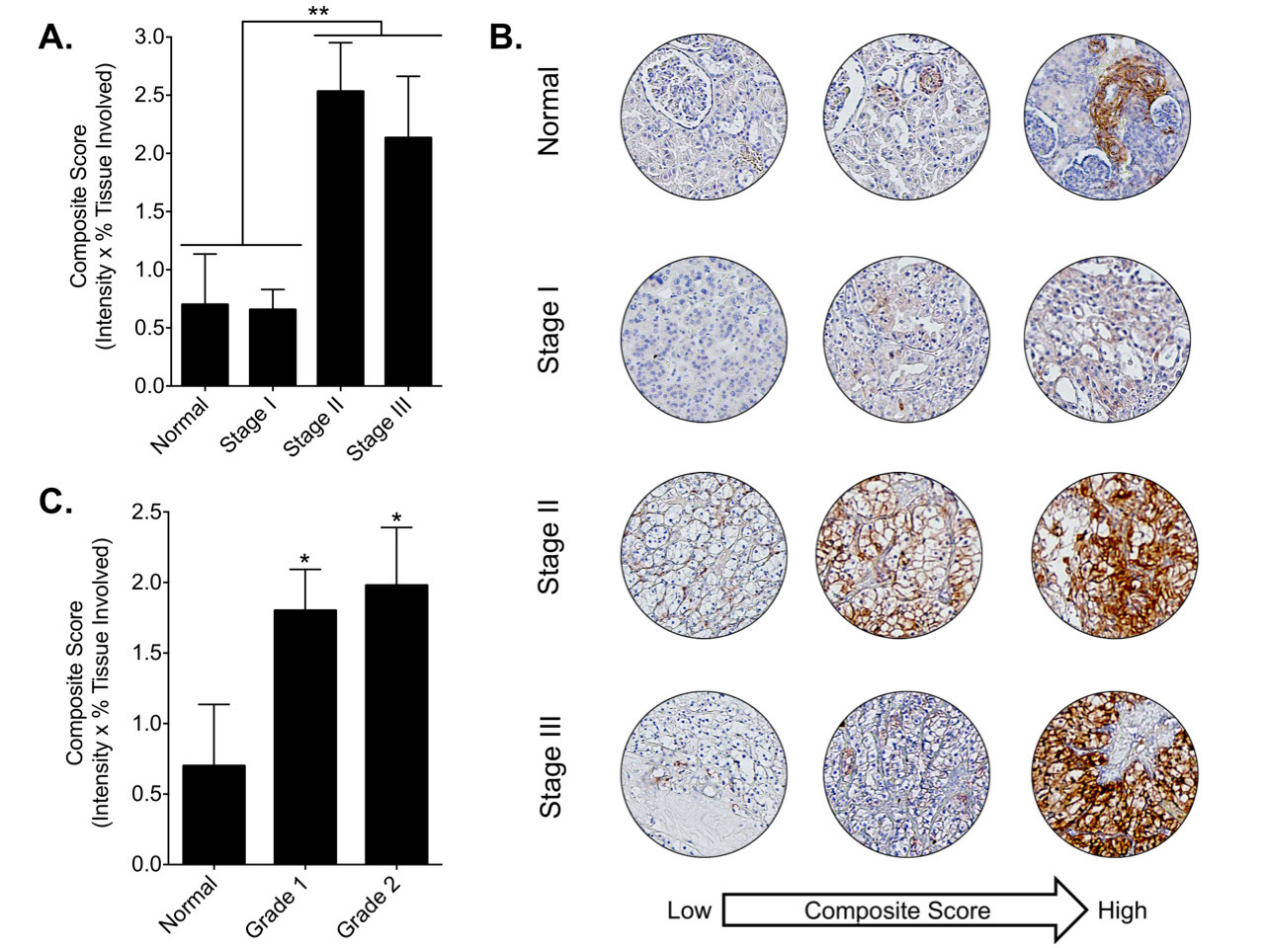
DCLK1 protein is overexpressed in RCC tumor tissue A) DCLK1 protein is significantly increased in stage II-III tumors compared to normal or stage I tumors (p<0.002). B) Representative DCLK1 immunoreactivity for normal, stage I, stage II, and stage III tissue samples from low to high composite score. C) DCLK1 immunoreactivity is elevated in grade 1 and grade 2 tumors.

To assess DCLK1 protein expression in RCC we performed immunohistochemistry using α-DCLK1 antibody on a commercially available tissue microarray. A chi-square test was performed to examine the relation between DCLK1 immunostaining and RCC diagnosis. The relation between these variables was significant, *X^2^*(1, *n* = 192) = 4.156, *p* <0.05, indicating that RCC tumors were significantly more likely to demonstrate DCLK1 immunostaining. Mean tumor expression of DCLK1 protein was 2 fold higher in tumors compared to normal kidney (data not shown). Moreover, stage II and III tumors demonstrated significantly increased DCLK1 protein expression compared to both normal kidney and stage I tumors (Fig 3A-B), and DCLK1 protein expression was also upregulated in grade I and II tumors (Fig 3C). Together, these data demonstrate that DCLK1 is epigenetically altered and significantly overexpressed in RCC, and dysregulation of DCLK1 methylation and mRNA expression are interrelated. Moreover, immunohistochemical staining confirms these findings for DCLK1 protein in patient tumors and demonstrates increased expression in mid-to-advanced stage disease.

### RCC primary tumors display molecular hallmarks of EMT correlating to survival

The spread of disease in many cancers has been linked to the expression of EMT transcription factors, which lead to an invasive phenotype characterized by low expression of epithelial marker E-cadherin (CDH1) and high expression of mesenchymal markers Vimentin (VIM) and N-cadherin (CDH2). We found that the VIM and CDH2 were overexpressed while CDH1 mRNA was significantly downregulated in RCC tumors compared to matched normal adjacent tissue (p<0.0001, Fig S2A). In stage-wise comparisons we determined that VIM was significantly increased in Stage III and IV compared to Stage I tumor tissue (p<0.05, Fig S1D). No stage-wise effects were seen for CDH1 or CDH2 (data not shown). Interestingly, three of the five patients that displayed lower expression of DCLK1 mRNA in tumors compared to normal adjacent tissue were among the few patients that displayed high expression of CDH1.

VIM gene expression and loss of E-cadherin are prognostic markers for cancer-specific survival in renal cell carcinomas [25, 26]. In order to assess overall survival in the KIRC dataset relative to VIM gene expression and CDH1 gene and protein expression, we selected the top and bottom 25^th^ percentiles respectively for analysis. High VIM expression was a prognostic factor for poor overall survival as determined by the Log-Rank test (p=0.035) with a median survival difference of 813 days. However, the Wilcoxon test which is sensitive to survival differences at earlier time points did not indicate statistical significance, suggesting that the reduction in overall survival is not likely attributable to the modest stage-dependent increases in VIM expression (Fig S1D). Low CDH1 gene and protein expression were both prognostic factors for poor overall survival as determined by the Log-Rank test (p<0.005) as well as the Wilcoxon test (p<0.002, Fig S2B). Using RNA-Seq data the median survival difference between groups was 248 days, while for protein it was 761 days. These data link RCC to an EMT phenotype, confirm the previously reported use of these markers as prognostic indicators, and support targeting factors that modulate EMT as a therapeutic strategy to increase RCC survival.

### DCLK1 knockdown inhibits EMT and mesenchymal marker expression and reduces invasion

**Figure 4:**
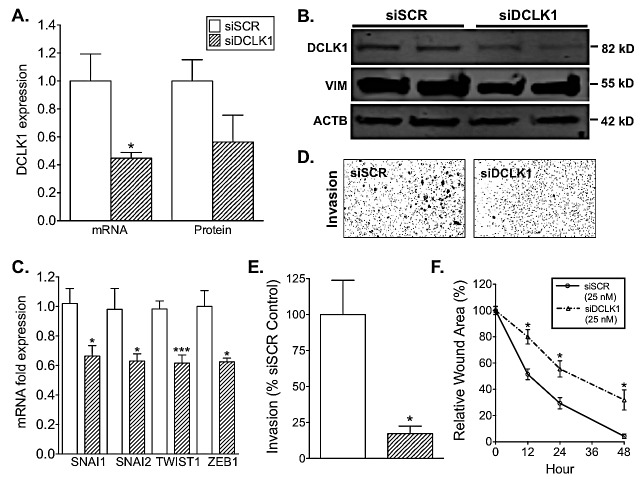
siRNA-mediated knockdown of DCLK1 reduces EMT factor expression, migration, and invasion in RCC cells A) siDCLK1 downregulates DCLK1 mRNA and protein expression relative to siSCR 48 and 72 h post transfection respectively in Caki-2 cells. B) siDCLK1 significantly decreases the expression of DCLK1 and mesenchymal marker vimentin 72 h post transfection relative to siSCR in Caki-2 cells. C) siDCLK1 downregulates EMT factor expression 48 h post transfection relative to siSCR in Caki-2 cells. D-E) siDCLK1 significantly decreases invasion relative to siSCR in Caki-2 cells (p<0.02). F) 25 nM siDCLK1 significantly reduces migratory capabilities relative to 25 nM siSCR transfected Caki-2 cells.

In order to assess the therapeutic potential of silencing DCLK1 in RCC we transfected Caki-2 cells with 25 nM DCLK1 siRNA (siDCLK1) targeting the primary DCLK1 protein-encoding transcripts (Fig S3A) or 25 nM siRNA of a scrambled sequence targeting no known genes (siSCR) as a control for 48 or 72 h. Following treatment 50-60% knockdown of DCLK1 was confirmed by real-time RT-PCR and Western blot (Fig 4A-B). Additionally, siDCLK1 treatment decreased cell proliferation by approximately 20% following 48 h transfection (Fig S3B).

DCLK1 has been shown to regulate the expression of EMT factors in multiple gastrointestinal cancer models [27-30]. Similarly, the expression of EMT transcription factors SNAI1, SNAI2, TWIST1, and ZEB1 was significantly decreased 48 h post siDCLK1 transfection (Fig 4C). While mesenchymal marker Vimentin protein expression was decreased 40-50% 72 h post transfection (Fig 4B). Based on the loss of EMT marker expression following siDCLK1 treatment, we assessed the functional effect of DCLK1 knockdown on RCC cell migration and invasion *in vitro*. There was an approximately 30% decrease in wound-healing capacity in Caki-2 cells treated with 25 nM of siDCLK1 compared to 25 nM siSCR controls at 12, 24, and 48 h post-wound formation (p<0.0003 for all points; Fig 4F). DCLK1 knockdown has previously been shown to reduce the invasive capabilities of pancreatic cancer cells [12]. 48 h post transfection Caki-2 invasion was reduced 80-90% in 25 nM siDCLK1 treated cells compared to 25 nM siSCR treated cells (p<0.02; Fig 4D-E). These results demonstrate that DCLK1 knockdown reduces the invasive and metastatic capabilities of RCC.

### DCLK1 knockdown compromises focal adhesion in RCC

**Figure 5:**
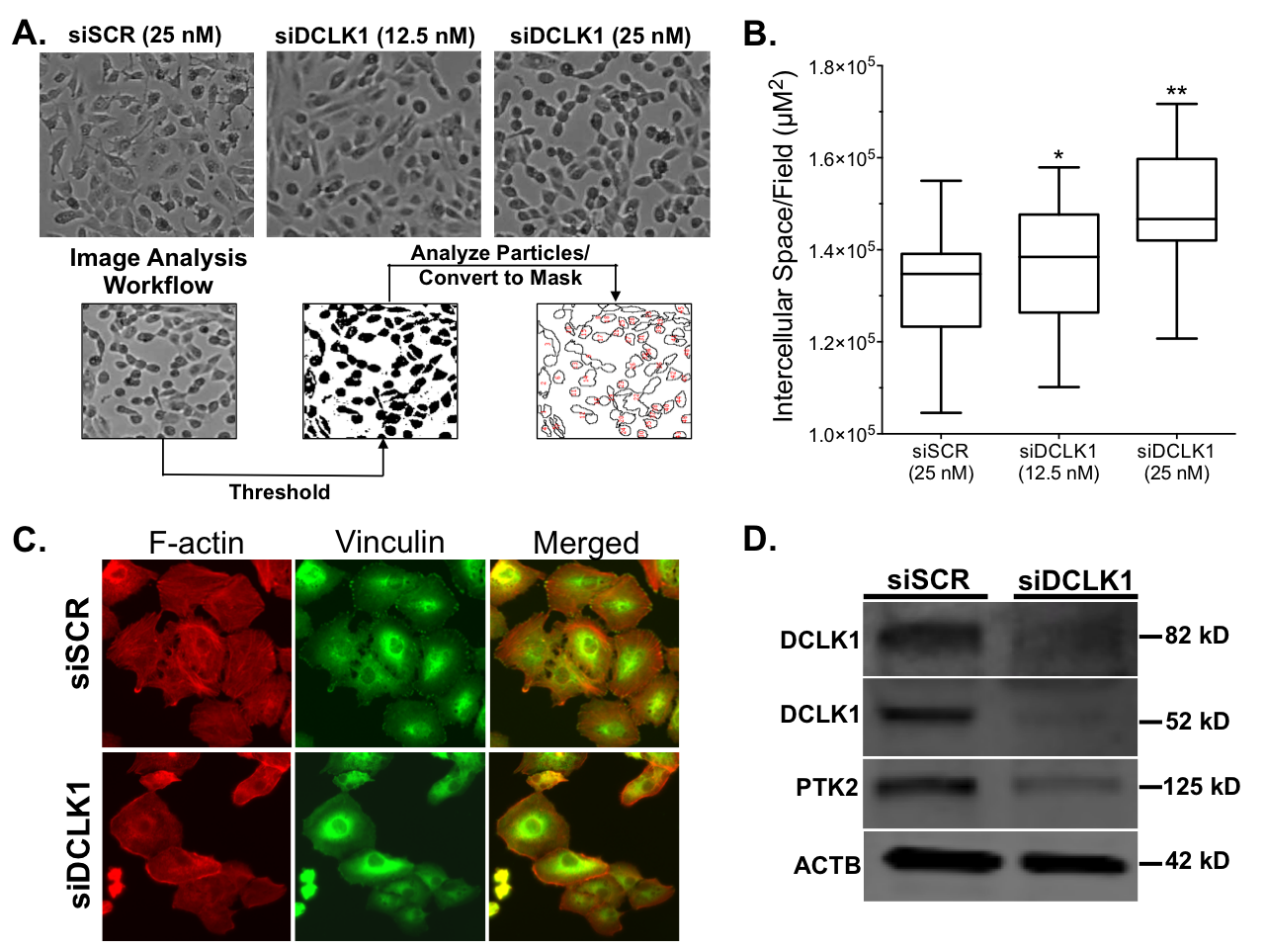
siRNA-mediated knockdown of DCLK1 reduces cell adhesion in RCC cells A) Representative images of dose dependent increase in intercellular space following 48 h siRNA transfection in Caki-2 cells and image intercellular space analysis workflow. B) Quantification of dose-dependent increase in intercellular space from image analysis (p<0.011). C) Immunocytochemistry demonstrating loss of rigid F-actin strands as demonstrated by TRITC-phalloidin staining and decreased focal adhesions (marked by α-vinculin) in siDCLK1 transfected Caki-2 cells compared to siSCR. D) Immunoblotting demonstrating a decrease in PTK2 (FAK) following siDCLK1 transfection of Caki-2 cells after 72 h.

During our studies it became apparent that cells transfected with siDCLK1 for 48 h appeared to demonstrate a uniform increase in the space between cells compared to siSCR treated cells. To quantify the observed increase in intercellular space, image-processing techniques were used to compare the unoccupied intercellular area between treatment groups (Fig 5A) and demonstrated a statistically significant increase in unoccupied area between both 12.5 nM and 25 nM siDCLK1 treated cells compared to 25 nM siSCR treated cells (p<0.011;Fig 5B). To rule out changes in cell cycle dynamics induced by siDCLK1 as a source of the aberrant adhesion we performed cell cycle analysis of 48 h siSCR and siDCLK1 treated Caki-2 cells. No notable changes in cell cycle were observed between the controls and treated cells (Fig S3C). Moreover, a significant decrease in PTK2 (FAK) protein expression, a key regulator of focal adhesion which network analysis suggests may be regulated through DCLK1′s physical association with calmodulin [31-33], was found 72 h post-transfection (Fig 5D; Fig S3D).

To confirm the decreased adhesive phenotype following siDCLK1 transfection, immunocytochemistry was performed using TRITC-phalloidin, which detects filamentous actin (F-actin), and vinculin antibody, which detects the specific sites of focal adhesions. A loss of rigid F-actin strands as well as a modestly decreased number of focal adhesions was observed in cells transfected with 25 nM siDCLK1 (Fig 5C). These results demonstrate a new functional role for DCLK1 in maintaining focal adhesion in cancer and another potential mechanism of action for siDCLK1′s anti-migratory and anti-invasive effects.

### DCLK1 knockdown decreases RCC stemness

**Figure 6:**
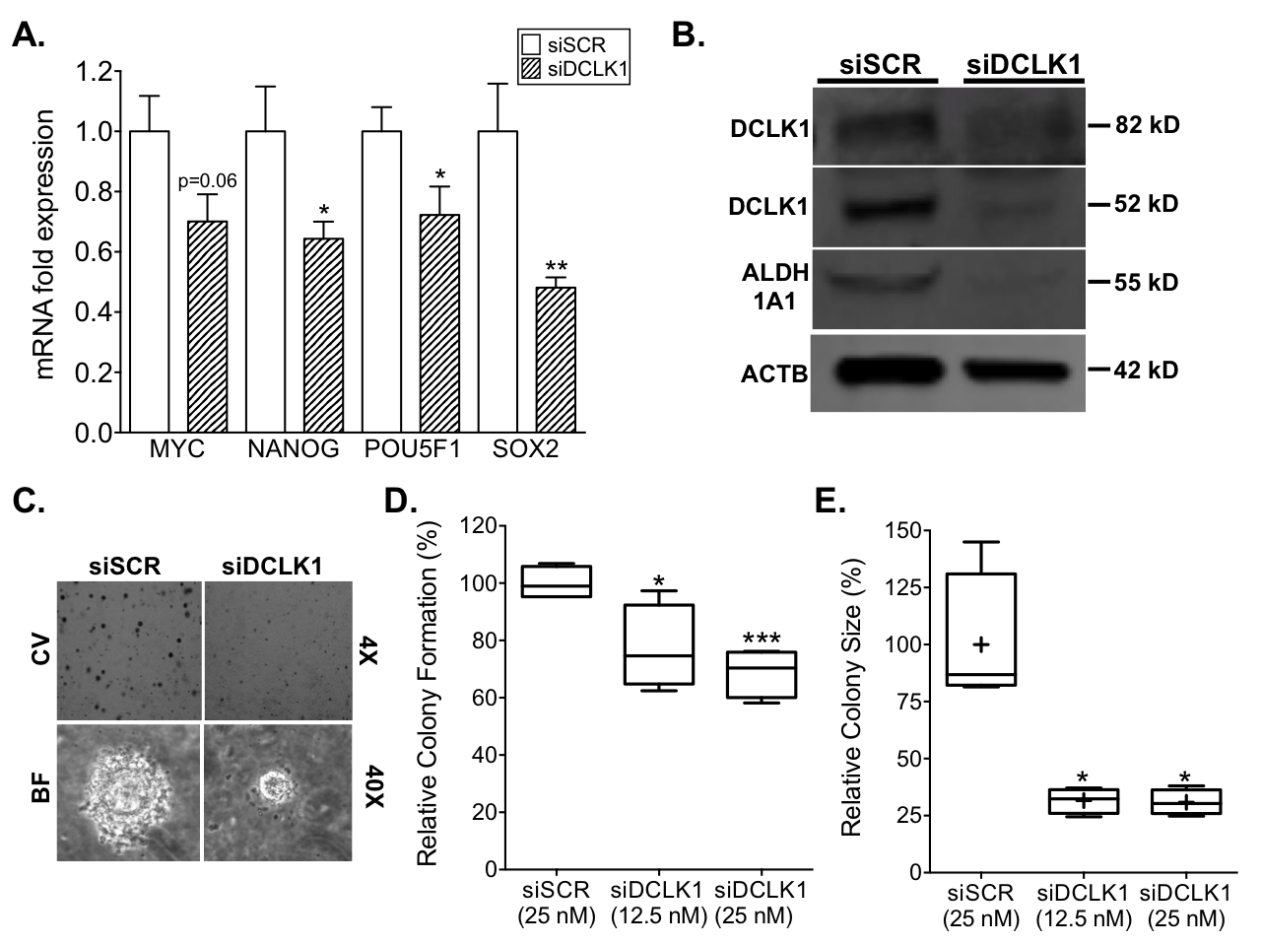
siRNA-mediated knockdown of DCLK1 inhibits pluripotency, drug-resistance, and clonogenic capacity in RCC cells A) siDCLK1 transfection downregulates the expression of pluripotency factors MYC, NANOG, POU5F1 (OCT4), and SOX2 in Caki-2 cells 48 h post transfection. B) siDCLK1 downregulates the ALDH1A1 putative RCC stem cell marker 72 h post transfection in Caki-2 cells. C-E) 48 h siDCLK1 pre-transfected Caki-2 cells demonstrate a significant decrease in clonogenic capacity (p=0.0009 and p=0.028 for 25 and 12.5 nM siDCLK1 transfection) and colony size (p=0.004) in a 0.3% soft agar assay (CV = crystal violet; BF = Brightfield).

As we have seen in other cancers and cancer cell lines [12], knockdown of DCLK1 reduced the expression of stem cell pluripotency factors MYC, NANOG, POU5F1/OCT4, and SOX2 in Caki-2 cells (Fig 6A). Moreover, the expression of ALDH1A1 (Fig 6B), a putative RCC stem cell marker that is associated with decreased recurrence-free and overall survival [34, 35], was significantly decreased 72 h post transfection. These findings demonstrate that DCLK1 regulates the expression of stemness and pluripotency related factors in RCC.

To investigate the alteration of stemness further, a clonogenic assay was performed with Caki-2 cells transfected with siDCLK1 or siSCR. Following 2 weeks of growth there was an approximately 25 and 30% decrease in the number of colonies formed from 12.5 nM and 25 nM siDCLK1 treated cells respectively relative to 25 nM siSCR treated cells. Additionally, there was a stark, approximately 70% decrease in the mean size of 12.5 and 25 nM siDCLK1 transfected colonies suggesting that DCLK1 is an important factor in RCC anchorage-independent growth (Fig 6C-E). These results as a whole demonstrate that DCLK1 is directly or indirectly responsible for maintaining functional stemness in RCC cells and suggest that disruption or inhibition of DCLK1 should be investigated as both a primary and adjuvant therapy in RCC.

## DISCUSSION

Research into RCC has not been pursued vigorously because nephrectomy is an efficient treatment for disease localized to the primary tumor, which seldom presents bilaterally [36]. However, local or distant spread significantly limits treatment options and decreases lifespan, and there is a desperate need to identify novel therapeutic targets that can slow or prevent this spread in order to improve the survival of patients with advanced disease. Moreover, RCC's survival-related EMT phenotype, hypoxic conditions, slow growth rate, and intense radio/chemo-resistance suggest that it may represent a model tumor stem cell driven cancer.

In this study we leveraged data generated by TCGA to demonstrate that DCLK1 is epigenetically dysregulated and overexpressed in RCC compared to normal tissue and confirmed these findings at the protein level by immunohistochemistry. Furthermore, we demonstrated that DCLK1 methylation and mRNA expression can serve as RCC tissue biomarkers, and confirmed a relationship between DCLK1 methylation and mRNA expression. Analysis of the TCGA methylation data for DCLK1 exemplifies the complex epigenetic alterations present in cancerous tissue and suggests the need to take a careful approach to analyzing these alterations in genes with multiple protein coding transcripts such as DCLK1. However, we are confident that with an understanding of these complex sets of alterations – highly specific DCLK1 methylation biomarkers can be identified.

One of the most novel findings of this study is that DCLK1 knockdown leads to a significant loss of cell adhesion, which may be integral to the anti-migratory and anti-invasive effects observed. It is well known that DCLK1 colocalizes with both microtubules and the actin cytoskeleton, but these associations and their significance in cancer have not been investigated in depth. Moreover, the observed loss of adhesion is also interesting in light of DCLK1′s roles in neurite formation and elongation [37] and should be investigated further in other cancers known to express DCLK1.

**Figure 7:**
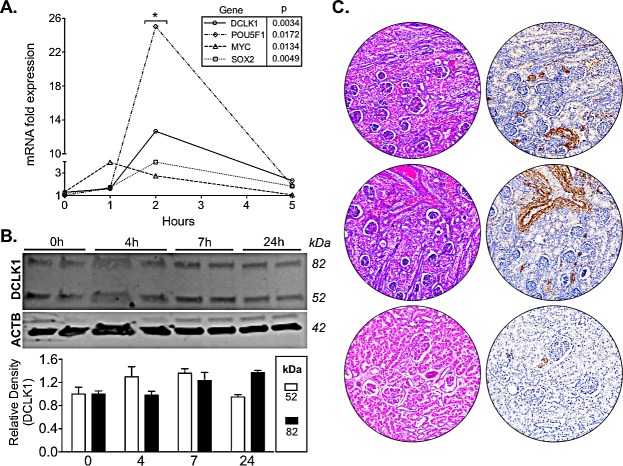
DCLK1 is induced under hypoxic conditions in RCC cells and is expressed in normal kidney A) DCLK1 mRNA expression is significantly induced in a time-dependent manner in tandem with pluripotency factors POU5F1 (OCT4), MYC, and SOX2 in RCC cells under 1% O_2_/5% CO_2_. B) Long and short-form DCLK1 protein expression is induced following 4 - 24 h under 1% _2_/5% CO_2_. C) DCLK1 protein is expressed in tubules, stroma, and endothelia of the normal kidney as demonstrated by IHC using α-DCLK1 antibody.

Given the importance of hypoxia and hypoxia-inducible factors in RCC we subjected Caki-2 cells to hypoxia to assess DCLK1 expression. Following 2 h of hypoxia we found that DCLK1 mRNA expression was massively increased along with pluripotency factors MYC, POU5F1/OCT4, and SOX2 (Fig 7A). Additionally, we found that DCLK1 protein expression was highly increased following 7 h of hypoxia (Fig 7B). These findings merit further study into DCLK1′s role in the hypoxic RCC tumor environment. In previous studies we have demonstrated that DCLK1 regulates angiogenic factors, which are tightly regulated by hypoxia inducible factors [38]. Further studies will be needed to assess whether inhibition or downregulation of DCLK1 is sufficient to reduce hypoxia inducible factor signaling, angiogenesis, and stemness in RCC under hypoxia.

Although a previous report using northern blotting indicated that DCLK1 was not expressed in the normal human kidney [39], we observed positive staining in renal tubules, stroma, and endothelia in normal sections present on the tissue microarray (Fig 7C). To our knowledge this will be the first reported data demonstrating DCLK1 expression in the normal kidney. Larger tissue sections from whole normal kidney will be needed for detailed characterization of DCLK1′s expression in this organ. However, we believe that this data in addition to the functional similarities between renal tubules and the small intestinal epithelium merits further investigation of DCLK1 in kidney diseases in addition to cancer.

Finally, we note that different therapeutic interventions targeting DCLK1 in cancer are currently under development. Since EMT is a major factor in the survival of RCC patients and DCLK1 regulates this process, some of these interventions may have the potential to significantly extend survival. Notably, a number of DCLK1 targeting agents have been developed in the kinase inhibitor class of drugs [29, 30] – a class that has proven effective in extending the lives of patients with advanced RCC. Moreover, our preliminary studies indicate that DCLK1 knockdown sensitizes RCC cells to receptor tyrosine kinase inhibitor sunitinib (Fig S3E), which may be beneficial in early treatment or extend sunitinib's efficacy after resistance has developed. This effect is likely related to the loss of stemness following DCLK1 knockdown. In addition, DCLK1 is overexpressed in a majority of patient tumors and has a targetable cell-surface domain. These characteristics suggest that it may be an optimal beacon for promising, targeted anticancer drugs such as monoclonal antibodies or antibody-drug conjugates. Further studies in animal models of RCC as well as patient derived xenograft and orthotopic models will be needed to determine the efficacy of these DCLK1-targeted therapeutics.

## MATERIALS AND METHODS

### Analysis of NCBI GEO and TCGA patient data

The standard data run for 11/14/2013 of The Cancer Genome Atlas' (TCGA) Kidney Renal Clear Cell Carcinoma (KIRC) dataset was downloaded from the Broad Institute's Genome Data Analysis Center using the firehose_get tool. Data was sorted and manipulated for analysis using the R statistical environment (version 3.0.2). NCBI Gene Expression Omnibus (GEO) data was downloaded directly.

### Pathological characterization of RCC tissue

A kidney cancer tissue microarray (US Biomax KD2085) containing 192 microsections including 20 normal kidney samples and 64 stage I, 76 stage II, 30 stage III, and 2 stage IV RCC samples was stained with hematoxylin and eosin and immunostained with α-DCLK1 antibody (ab31704) following a previously described immunohistochemistry protocol [29]. Each stained tissue micro-section was scored independently by two pathologists and based on percent of tissue demonstrating staining (1 for <10% - 4 for > 60%) and staining intensity (1 for lowest intensity - 4 for highest intensity). The resulting scores were multiplied by each other to obtain a composite score.

### siRNA-mediated knockdown of DCLK1

Caki-2 human clear cell renal carcinoma cells were purchased from the American Type Culture Collection (ATCC) and maintained in RPMI medium containing 10% fetal bovine serum (FBS). For siRNA-mediated knockdown studies, 10^5^ Caki-2 cells were seeded into 6 cm petri dishes and allowed to attach overnight. Following attachment, 25 nM of commercially validated siRNA targeting human DCLK1 exons 4/8 of the β/α-promoter isoforms respectively (siDCLK1; Life Technologies s17584) or 25 nM human scrambled sequence (siSCR) not targeting any known genes were complexed with Lipofectamine 3000 (Invitrogen) and added to the dishes in fresh cell culture medium. After 48 and 72 h of treatment respectively, RNA and protein were collected for analysis.

### Quantitative real-time RT-PCR

Total RNA was isolated from Caki-2 cells using Tri Reagent (MRC) per the manufacturer's instructions. First strand cDNA synthesis was carried out using SuperScript II Reverse Transcriptase and random hexanucleotide primers (Invitrogen). The complementary DNA was subsequently used to perform RT-PCR on an iCycler IQ5 Thermal Cycler (BioRad) using SYBR Green (Molecular Probes) with gene-specific primers and JumpStart™ Taq DNA polymerase (Sigma). The crossing threshold value assessed was normalized to β-actin and quantitative changes in mRNA were expressed as fold-change relative to control ± SEM value. The Student's t-test was used to determine statistical significance. The primer sequences for the genes analyzed are provided in Table SII.

### Western blotting

Denatured proteins of cell lysates were subjected to Western blot analysis. The concentration of total proteins was determined by BCA protein assay (Pierce, Rockford, IL). 40 μg of total proteins was separated on a 7.5%–15% SDS polyacrylamide gel and transferred onto a PVDF membrane. The membrane was blocked in 5% non-fat dry milk for 1 h and probed overnight with primary antibody. Subsequently the membrane was incubated with infrared cw800-conjugated secondary antibody for 1 h at room temperature. The proteins were detected using a LICOR Odyssey Infrared Imager. Protein density quantification was performed in Image Studio Lite (LICOR).

### Proliferation Assays

For analysis of siDCLK1′s effect on proliferation 10^5^ Caki-2 cells/well were seeded into 2 six well plates. Each plate was transfected with 25nM siDCLK1 or siSCR respectively for 48 h after which resazurin (alamar blue; Biotium) was added to each well according to the manufacturer's protocol. After 2 h the fluorescence was read at 590 nm. For calculations, values were normalized to siSCR.

### Wound healing assay

10^5^ Caki-2 cells/well were seeded into 6-well plates, allowed to grow to 50% confluence, and transfected with 25 nM siDCLK1 or 25 nM siSCR for 48 h. Following a 24 h recovery period, a sterile 200 μl pipette tip was used to scratch the confluent layer, and detached cells were removed with washing. For each plate 9 points were selected and marked and baseline images were taken at 4x magnification. Images were again taken at 12, 24, and 48h. Images were converted to 16-bit format, thresholded, and the migrating edges were detected using the *analyze particles* function in ImageJ. All replicates were normalized to their baseline value and migration was reported as relative wound area remaining.

### Analysis of Focal Adhesion

Level of focal adhesion was qualitatively assessed by immunocytochemistry using an actin cytoskeleton/focal adhesion staining kit (Millipore) containing TRITC-conjugated phalloidin and mouse α-Vincullin according to the manufacturer instructions. To increase clarity cell nuclei were counterstained with Hoechst 33342. In order to quantitatively assess cell adhesion images of siDCLK1 or siSCR transfected cells (48 h transfection + 24 h recovery) were taken at 4X at 1280 × 960 resolution where 1 pixel is equivalent to 1 μM. The images were converted to 16-bit, thresholded, and assessed with the *analyze particles* function in ImageJ. The sum of the area of all particles (cells) combined was subtracted from the total image area to give the area of unoccupied intercellular space. These experiments were carried out twice independently to ensure precision.

### Matrigel transwell invasion assay

Matrigel coated transwells (BD Biosciences) were prepared by soaking in serum-free media for 2 h at 37ºC in a 24-well plate. Subsequently, Caki-2 cells (5000/well) pre-transfected with either 25 nM siDCLK1 or siSCR for 48 h were seeded into each transwell in serum-free media in triplicate. Cell culture medium containing 10% FBS was added to the bottom of each well as chemoattractant and the cells were incubated for 22h at 37°C under 5% CO_2_. Afterwards, a cotton swab was used to scrape non-invasive/migratory cells off the top of transwells and the remaining cells were fixed with 100% methanol, stained with 0.1% crystal violet, and allowed to dry. After drying all invading cells were counted from each transwell at 4x magnification. Cell number was normalized to siSCR control x 100 and results were reported as invasion (%siSCR Control).

### Colony formation assay

Caki-2 cells were seeded into a 6 cm dish and transfected with 25 nM siSCR, 12.5 nM siDCLK1, or 25 nM siDCLK1 using Lipofectamine 3000 as a transfection reagent. Following 48 h transfection, 5000 Caki-2 cells/well (4 replicates per siRNA treatment) were seeded into 0.3% soft agar on top of a 0.6% soft agar base in a 24 well plate. 250 ul of complete medium was added on top of each well and replenished every 3^rd^ day. The cells were allowed to form colonies for 14 days and on day 14 the plates were stained with 0.5% crystal violet. A 4X image was taken of each well and analysis was performed by using a macro to automatically convert images to 16-bit, threshold, and run the *analyze particles* function to count the number of cells and measure the area of each. The collected data were normalized to siSCR and reported as relative percent change.

### Induction of Hypoxia in RCC Cells

10^5^ Caki-2 cell/well were seeded into a 6-well plate and allowed to attach overnight. Following attachment cells were incubated either under normoxia or hypoxia (1% O_2_/5% CO_2_). Cells were lysed at various time intervals and subjected to mRNA or protein expression analysis as described.

### Statistical Analyses

All statistical analyses were performed in Graphpad Prism 6.0, SPSS Statistics 22, and Microsoft Excel. For non-parametric data the Mann-Whitney U test was used, and for parametric data the Student's tTest was used. For survival analyses the Log-rank (Mantel-Cox) test and the Gehan-Breslow-Wilcoxon test were used. A p-value of less than 0.05 was considered statistically significant for all analyses.

## SUPPLEMENTARY MATERIAL, FIGURES, TABLES


