# Supplementary material for: DCLK1 is a broadly dysregulated target against epithelial-mesenchymal transition, focal adhesion, and stemness in clear cell renal carcinoma
Source: Oncotarget. 2014 Dec 11;6(4):2193–205. doi: 10.18632/oncotarget.3059 (PMC4385845; doi:10.18632/oncotarget.3059)
Supplement: Supplementary file 1 [file oncotarget-06-2193-s001.pdf]

DCLK1 is a broadly dysregulated target against epithelial-mesenchymal transition, focal adhesion, and stemness in clear cell renal carcinoma

Supplementary Material

Supplementary Table I. Clinical Characteristics of TCGA KIRC Patients Studied.

Parameter	Males	Females	Overall
**Sample Size**	330	173	503
**Mean Age**	59.4	62.9	60.6
**Median Age**	59	64	61
**95% C.I. (Age)**	58.1 - 60.7	61.1 - 64.8	59.5 - 61.7
**% Metastatic**	16.67%	13.29%	15.51%
**% Fatal**	31.51%	35.26%	32.80%
**Ethnicity**			
White	306	152	458
Black/African	20	17	37
Asian	4	4	8

Supplementary Table II. Primers Used in Real Time RT-PCR Experiments.

Gene ID	Forward (5' – 3')	Reverse (5' – 3')
MYC	CACACATCAGCACAACTACGCA	TTGACCCTCTTGGCAGGCAG
DCLK1	TAGCCAGCGCCATCAAATAC	ACCCAGCTTCAGTGATTTGC
TWIST1	GCCGGAGACCTAGATGTCATTG	AGTTATCCAGCTCCAGAGTCTC
NANOG	ACCAGAACTGTGTTCTCTTCCACC	CCATTGCTATTCTTCGGCCAGTTG
SOX2	CGAGATAAACATGGCAATCAAAAT	AATTCAGCAAGAAGCCTCTCCTT
SNAI1	GCCATGTCCGGACCCACACTG	GGCAGGGGCAGGTATGGAGA
ZEB1	AAGAATTCACAGTGGAGAGAAGCCA	CGTTTCTTGCAGTTTGGGCATT
ACTB	GCTGATCCACATCTGCTGG	ATCATTGCTCCTCCTGAGCG
POU5F1	AAGCGATCAAGCAGCGACTAT	GGAAAGGGACCGAGGAGTACA
SNAI2	AAGGCCTTCTCTAGGCCCT	CGCAGGTTGAGCGGTCAG

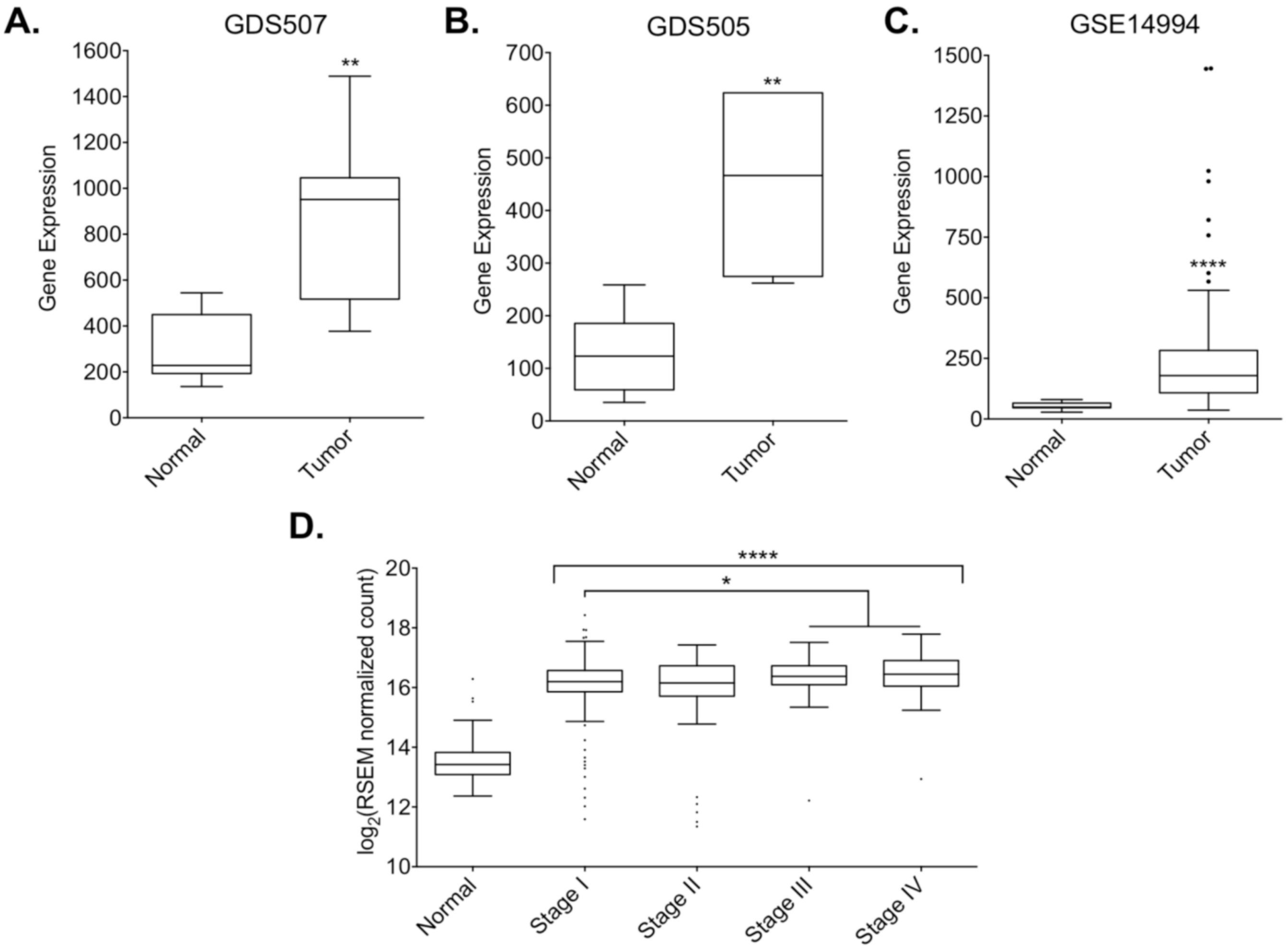

Supplementary Figure 1. A-C) The DCLK1 gene is overexpressed in RCC microarray data from the NCBI gene expression omnibus. **D)** Vimentin is overexpressed in RCC and demonstrates a signficant increase in stages III-IV compared to stage I.

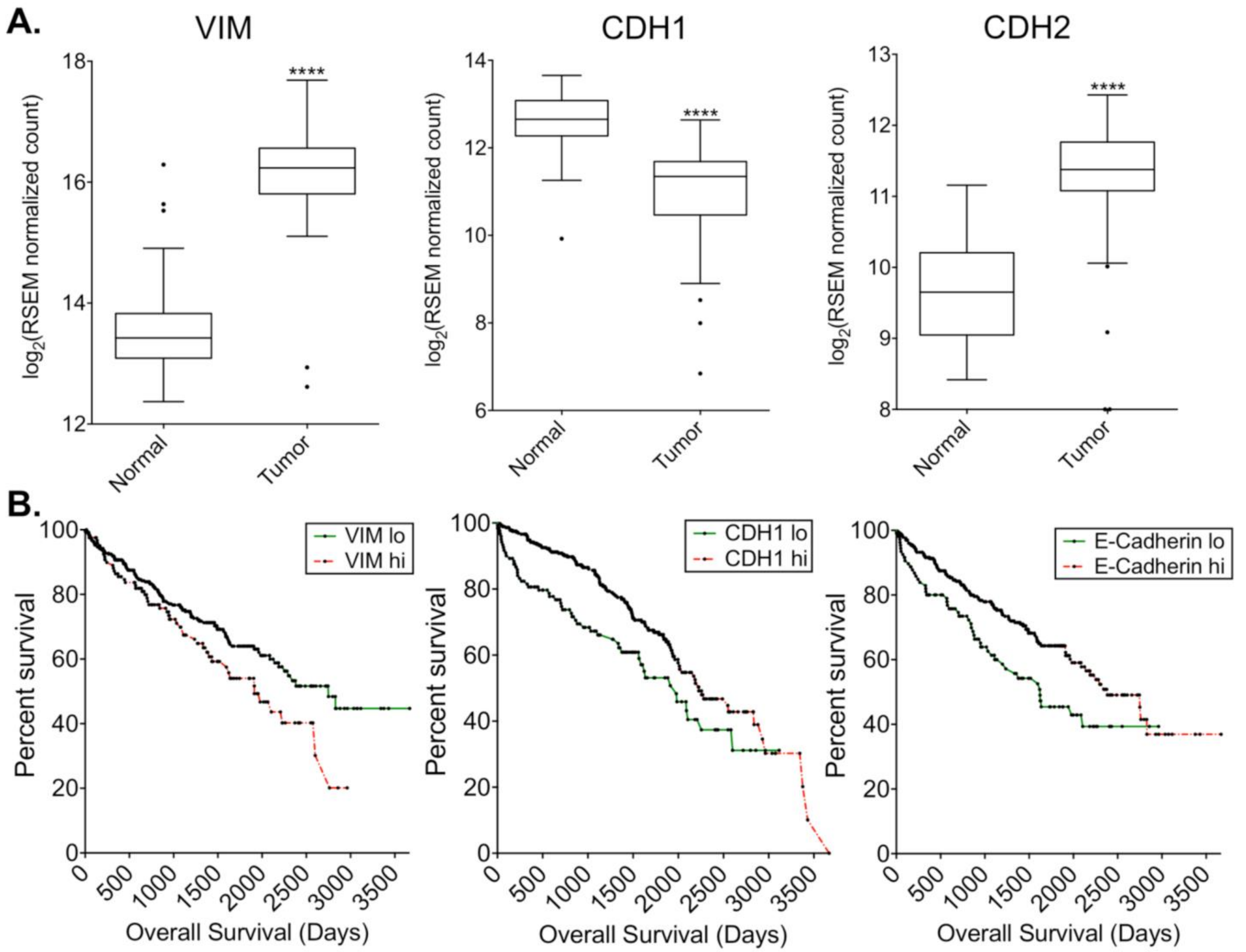

Supplementary Figure 2. A) Mesenchymal gene expression (VIM and CDH2) is upregulated and epithelial gene expression (CDH1) is downregulated in RCC. **B)** High VIM gene expression and low CDH1/E-cadherin gene and protein expression are correlated to significantly decreased survival in RCC.

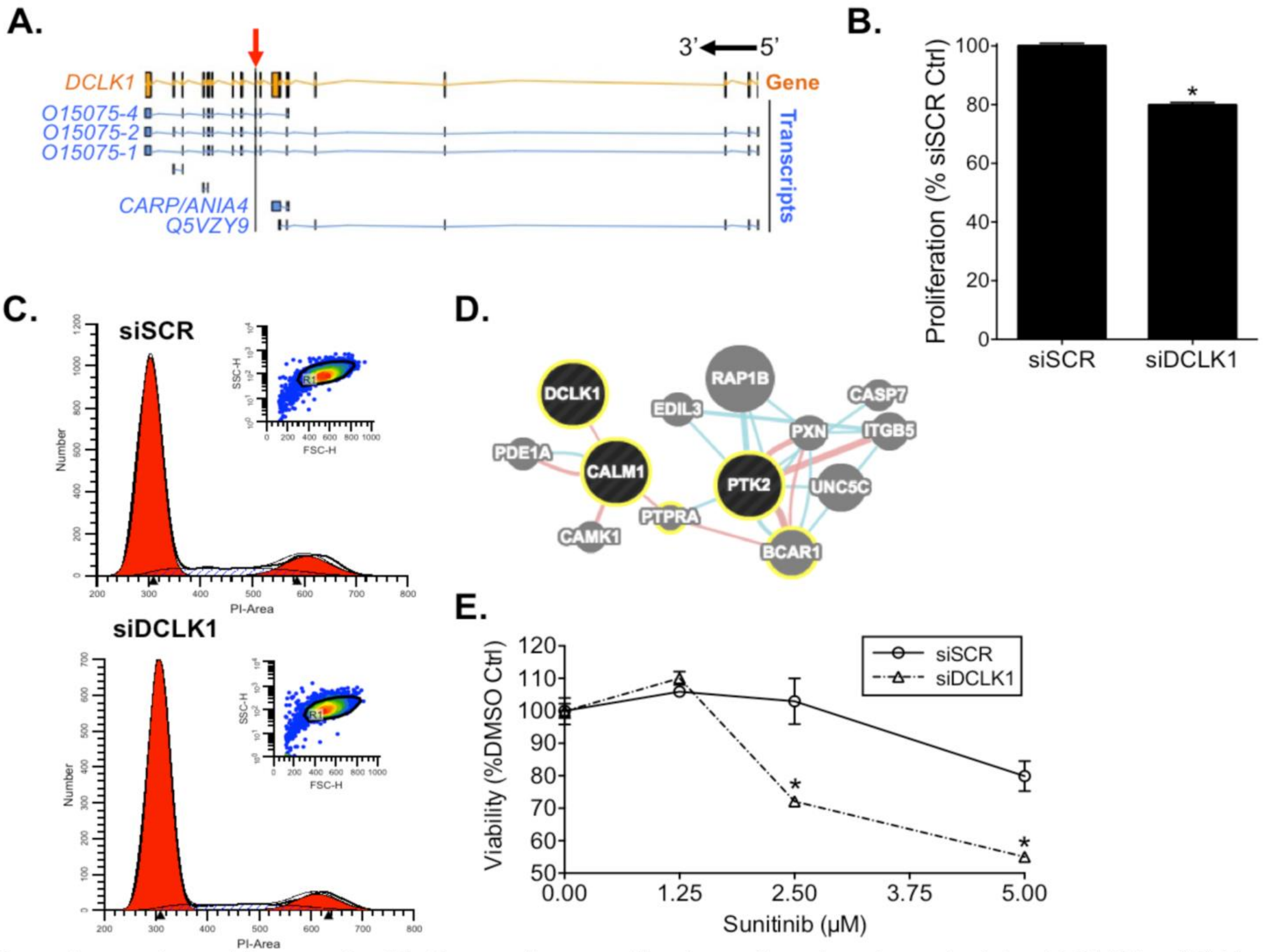

Supplementary Figure 3. A) Genomic coordinates of region targeted by DCLK1 siRNA. **B)** siDCLK1 transfection significantly reduces Caki-2 cell proliferation relative to siSCR transfection. **C)** siDCLK1 transfection does not alter the cell cycle status of Caki-2 cells. **D)** Genemania network analysis of DCLK1's interaction with PTK2/FAK demonstrating that DCLK1 physically interacts with calmodulin-1 (CALM1), which physically interacts with PTK2/FAK through PTPRA. **E)** To investigate whether siDCLK1 sensitizes RCC cells to receptor tyrosine kinase inhibitors, Caki-2 cells pretreated with either siDCLK1 or siSCR were seeded into a 96-well plate and treated with DMSO (vehicle), 5, or 10 μM sunitinib for 48 h. Following treatment a proliferation assay was performed and demonstrated that siDCLK1 sensitizes Caki-2 cells to sunitinib ($p<0.0002$).
